# Pain Flare After Single Fraction Radiotherapy for Symptomatic Bone Metastases—Do We Have a Predictive Model?

**DOI:** 10.1002/cam4.71175

**Published:** 2025-08-21

**Authors:** Tatjana Arsenijević, Aleksandar Stepanović, Snježana Bogićević, Dragica Gržetić, Marina Nikitović

**Affiliations:** ^1^ Faculty of Medicine University of Belgrade Belgrade Serbia; ^2^ Institute for Oncology and Radiology of Serbia Belgrade Serbia

**Keywords:** bone metastases, pain flare, prediction, radiotherapy

## Abstract

**Background:**

The COVID‐19 pandemic reintroduced single‐fraction radiotherapy for symptomatic bone metastases due to its efficiency, rapid realization, and cost‐effectiveness. However, pain flare can occur as a side effect that can have a significant impact on a patient's quality of life. With pain flare incidence ranging between 2% and 44%, there are no definitive conclusions about which patients are at greater risk.

**Aim:**

This study aims to analyze the occurrence of pain flare in relation to age, patient's performance status, primary tumor, histopathology, and bone localization of metastases.

**Methods:**

Our clinical, prospective, non‐randomized study included 64 patients with non‐complicated, painful bone metastases who underwent palliative, pain‐relieving 8 Gy single‐fraction radiotherapy in a single hospital visit. Response to treatment was patient‐reported via telephone interview using a visual analog scale and assessed based on the international consensus panel of radiation oncologists.

**Results:**

In the entire group of patients, 17% developed pain flare. No statistically significant difference was observed in pain flare occurrence depending on the patient's age, performance status, the primary origin of the tumor, histopathology, or location of the metastasis (bone) that was irradiated. All but one patient had a good response to treatment after the flare.

**Conclusion:**

Pain flare can be expected in less than 20% of patients receiving 8 Gy single fraction radiotherapy for painful bone metastases. It is not proven that any of the mentioned clinical parameters influence the occurrence of pain flare. Pain flare does not interfere with response to treatment.

## 1 Introduction

Bone metastases are the most common cause of cancer‐related pain. It results in significant morbidity and severely affects patients' quality of life, often requiring prompt attention. The complicated pathophysiological mechanism of bone metastases and pain has not been fully clarified, leaving clinicians with a complicated choice of the best treatment modality for these patients [1, 2, 3, 4, 5, 6, 7, 8, 9, 10].

Radiotherapy has been the most effective therapeutic method for painful bone metastases for decades, with 80% of patients experiencing a reduction in pain and 25%–30% of patients experiencing complete pain relief. To achieve an analgesic effect, reduce the use of analgesics, and preserve the quality of life, numerous studies and meta‐analyses have tried to define the most optimal radiotherapy fractionation regimen. Equivalent pain response rates established palliative 8 Gy single‐fraction radiotherapy non‐inferior to the multi‐fraction regimen if pain control was the intent [11, 12, 13, 14, 15, 16, 17]. In the era of challenging selection of novel radiotherapy techniques, many authors still favor this simple single‐fraction external‐beam radiotherapy (EBRT) technique due to its efficiency, rapid realization, and thus, cost‐effectiveness. The whole paradigm of the fast approach found its approval during the COVID‐19 pandemic, suggesting that it may be favorable onwards [18, 19, 20, 21, 22].

Palliative radiotherapy for painful bone metastases is usually well‐tolerated. However, pain flare can occur as a side effect. Chow et al. defined pain flare as “temporary worsening of pain in the treated metastatic site” [23] that can have a significant impact on a patient's quality of life. Not only does it interfere with the patient's mobility, activity, and functioning, but it disturbs sleep and nutrition and provokes anxiety, resulting in what is described as “total pain” [24].

The mechanism of pain flare is largely unknown, so the management is left “at physician discretion” based mostly on increased analgesic use that can bring even more adverse events [24]. The theory of inflammation and periosteal edema caused by radiation highlighted corticosteroids as a potentially suitable pain flare treatment option. Available data also suggest that corticosteroid therapy (dexamethasone) may be beneficial as pain flare prophylaxis [25, 26, 27, 28].

Although several previous studies reported that patients who received radiation therapy with 8 Gy in one fraction may be at a higher risk for developing pain flare [29, 30, 31], it is known that multi‐fraction and stereotactic body radiotherapy (SBRT) can also cause pain flare as a side effect [23, 32, 33, 34]. Still, with the pain flare incidence ranging between 2% and 44% for EBRT and 10%–68% for SBRT in the literature, there are no definitive conclusions about which of them are at a greater risk for this phenomenon [33, 34]. Not only would the identification of these patients lead to a more personalized radiotherapy approach, but it could also lead to better optimization of multiple hospital visits for analgesic dose corrections, escalations, and toxicity, withdrawing patients from their everyday functioning and delaying further oncological treatment.

Therefore, our prospective, non‐randomized study aims to analyze the occurrence of pain flare in relation to age, performance status of the patient, primary tumor, histopathology, and localization of metastases in patients who were treated with 8 Gy single fraction radiotherapy for painful bone metastases. This group of patients was previously analyzed for response to treatment [35].

## 2 Methods

This clinical, prospective, non‐randomized study was conducted at the Institute for Oncology and Radiology of Serbia in the period from October 2019 to June 2020. All patients in this study were treated based on the current protocols for the diagnosis and treatment of malignant diseases, and the treatment was proposed by a multidisciplinary Tumor board. Written informed consent about the treatment protocol and participation in the study was obtained from all the patients.

### 2.1 Study Population

The study enrolled sixty‐four patients older than 18 years, with histopathologically proven any malignant disease, with radiographically and/or histopathologically confirmed bone metastases (one or more), previously treated or not treated with specific oncological therapy, and whose expected survival time was longer than 1 month. Patients with complicated bone metastases (spinal cord compression, pathological fracture), patients previously irradiated for the same bone metastases, and those with an expected survival time of less than 1 month were excluded from the study.

After the clinical examination with ECOG performance status estimation, pain intensity was assessed based on the visual analog scale (VAS), as well as the use of analgesics according to WHO recommendations [36, 37, 38].

### 2.2 Treatment Plan

All patients received palliative, pain‐relieving external‐beam radiation therapy with a tumor dose of 8 Gy in one fraction, with high‐energy photons (6 MeV, 8 MeV) on a linear accelerator. Radiation fields were determined individually for each patient based on reference bone X‐ray, CT, or MR images, and according to ICRU 62 recommendations [39].

### 2.3 Response to Treatment

Pain intensity, performance status, and the correction of analgesic use were evaluated first after 48 h following radiation, then once a week during the first 4 weeks, and then once a month until the third month after the radiation, using the same scales as before radiation. These assessments were carried out through telephone interviews or regular check‐ups, based on the standards of the international consensus panel of radiation oncologists [40].

Pain flare was defined as an increase in pain by ≥ 2 on the ordinal pain scale after irradiation, with spontaneous regression of pain over time or ≥ 25% increase of analgesic intake with no decrease in worst pain score.

None of the patients was given dexamethasone or bisphosphonates as pain flare prophylaxis (day 1–5 of radiation).

Pain flare management was individualized, based on pain intensity, performance status, patient comorbidities, and previous analgesics use. Analgesic changes/escalation, dexamethasone, or both were allowed.

The concurrent systemic treatment (systemic treatment within the same month) was not withdrawn.

For each patient in our study, follow‐up lasted 3 months from the date of irradiation or until the date of death.

### 2.4 Statistical Analyses

To examine the agreement of sample distributions with the normal distribution, graphs were used: Normal Q–Q Plot and Histogram, as well as tests: Kolmogorov–Smirnov and Shapiro–Wilk. For the description of the important parameters, and depending on their nature, measures of descriptive statistics were used: frequencies, percentages, mean value (average), median, standard deviation (SD), and range (range). The value *α* = 0.05 was adopted for the level of statistical significance. In case of multiple testing on the same data set, Bonferroni correction of *α*‐value was used. To test the differences between treatment groups, the Fisher exact test was used. Standard tests in the statistical program R version 3.3.2 (2016‐10‐31)—“Sincere Pumpkin Patch”; Copyright (C) 2016 The R Foundation for Statistical Computing; Platform: ×86_64‐w64‐mingw32/×64 (64‐bit) (Available at: www.r‐project.org; Retrieved: January 21, 2017) were used in Data processing and analysis.

## 3 Results

### 3.1 Patients Characteristics

The study included 64 patients older than 18 years of age with an average of 66 years (range 43–87 years). Almost half of the patients were ECOG PS 3 and 4 (34% and 8% respectively). Since the study included “any cancer” patients with painful bone metastases, the group was very heterogeneous. Still, 34% of patients had confirmed primary lung cancer, and in 33% of cases, histopathology was adenocarcinoma. Our patients predominantly had metastases in the spinal column (58%) and in the pelvis (25%). Other bone localizations were sporadic, but on the long bones side (femur, humerus). More than a third of patients (36%) had very strong pain (VAS 8–10), of which six (10%) had a severity of 10 on the VAS. Almost 40% of patients used strong opioids to control the pain syndrome.

The characteristics of the patients included in our study are presented in Table 1.

**TABLE 1:** Patients characteristics in the whole group (*N* = 64).

Age (years)		
40‐49	4	6.25%
50‐59	15	23.44%
60‐69	28	43.75%
70‐79	13	20.31%
80‐89	4	6.25%
Median (range)	66 (43–87)	
Sex		
Female	26	40.63%
Male	38	59.37%
Performance status (ECOG PS)		
1	18	28.12%
2	19	29.69%
3	22	34.38%
4	5	7.81%
Primary tumor localization		
Breast	5	7.81%
Colon	5	7.81%
Lung	22	34.38%
Cervix	2	3.12%
Unknown primary	4	6.25%
Hypopharynx	2	3.12%
Prostate	6	9.37%
Kidney	6	9.37%
Uterus	1	1.56%
Penis	2	3.12%
Bladder	4	6.25%
Esophagogastric	1	1.56%
Liver	2	3.12%
Rectum	2	3.12%
Histopathology	21	32.81%
Adenocarcinoma		
Squamous cell	9	14.06%
Small cell	7	10.94%
Unknown	7	10.94%
Ductal	3	4.69%
Renal cell	5	7.81%
Endometrial	1	1.56%
Transitional cell	6	9.37%
LCNEC	1	1.56%
NET	2	3.12%
HCC	1	1.56%
Lobular carcinoma	1	1.56%
Pain intensity before RT (VAS)		
No pain (0–2)	0	0.00%
Mild pain (3–4)	19	29.69%
Moderate pain (5–7)	22	34.38%
Severe pain (8–10)	23	35.94%
Analgesics		
No analgesics	7	10.94%
NSAID	21	32.81%
Weak opioides	11	17.19%
Strong opioides	25	39.06%
Localization of the metastases		
Pelvis	16	25.00%
Spinal vertebrae	37	57.81%
Humerus	6	9.37%
Femur	4	6.25%
Ribs	1	1.56%

Abbreviations: HCC, hepatocellular carcinoma; LCNEC, large cell neuroendocrinic tumor; NET, neuroendocrinic tumor; NSAIL, nonsteroid antiinflammatory drugs; RT, radiotherapy; VAS, visual analog scale.

### 3.2 Response to Treatment and Pain Flare

As previously reported [35], on an average of 3 weeks (range 0–13), 53 out of 64 patients (83%) responded to radiotherapy, while 11 patients (17%) did not respond. Of the 53 radiation respondents, 19 (36%) reached a complete response (complete absence of pain). The other 34 patients (64%) reached maximal partial response with a median difference in pain reduction of 4 points on the VAS (rank 3–6).

Pain aggravation after irradiation (pain flare) occurred in 11 of our patients (17%), while most of our patients (83%) did not have pain flare after 8 Gy single fraction radiotherapy.

In the group of patients who developed pain flare, there were seven male patients (64%) and four women (36%). The mean age was 66 years (range 53–77). More than half (six patients, 55%) were in ECOG PS1; three patients (27%) were in ECOG PS2, and only two (18%) were in ECOG PS3.

More than a third of the patients with pain flare (four patients, 37%) had primary lung cancer. Two patients (18%) had renal cell cancer; two gynecological tumors were reported, one prostate, one bladder cancer, and one unknown primary. In almost all of them, the metastases were localized in the spine and pelvis (Table 2).

**TABLE 2:** Patients characteristics in the „pain flare “group of patients (*N* = 11).

Age (years)		
40–49	0	0.00%
50–59	3	27.27%
60–69	6	54.55%
70–79	2	18.18%
80–89	0	0.00%
Median (range)	66 (53–77)	
Sex		
Female	4	36.36%
Male	7	63.64%
Performance status (ECOG PS)		
1	6	54.55%
2	3	27.27%
3	2	18.18%
4	0	0.00%
Primary tumor localization		
Breast	0	0.00%
Colon	0	0.00%
Lung	4	36.36%
Cervix	1	9.09%
Unknown primary	1	9.09%
Hypopharynx	0	0.00%
Prostate	1	9.09%
Kidney	2	18.18%
Uterus	1	9.09%
Penis	0	0.00%
Bladder	1	9.09%
Esophagogastric	0	0.00%
Liver	0	0.00%
Rectum	0	0.00%
Histopathology		
Adenocarcinoma	2	18.18%
Squamous cell	1	9.09%
Small cell	1	9.09%
Unknown	1	9.09%
Ductal	0	0.00%
Renal cell	2	18.18%
Endometrial	1	9.09%
Transitional cell	1	9.09%
LCNEC	1	9.09%
NET	1	9.09%
HCC	0	0.00%
Lobular carcinoma	0	0.00%
Pain intensity before RT (VAS)		
No pain (0–2)	0	0.00%
Mild pain (3–4)	3	27.27%
Moderate pain (5–7)	3	27.27%
Severe pain (8–10)	5	45.45%
Analgesics		
No analgesics	1	9.09%
NSAID	4	36.36%
Weak opioides	1	9.09%
Strong opioides	5	45.45%
Localization of the metastases		
Pelvis	4	36.36%
Spinal vertebrae	6	54.55%
Humerus	1	9.09%
Femur	0	0.00%
Ribs	0	0.00%

Abbreviations: HCC, hepatocellular carcinoma; LCNEC, large cell neuroendocrinic tumor; NET, neuroendocrinic tumor; NSAIL, nonsteroid antiinflammatory drugs; RT, radiotherapy; VAS, visual analog scale.

Out of the 11 patients who had a pain flare, five of them initially had very severe pain (8 on the VAS), and three had moderate pain (5–7 on the VAS). In three patients, the pain was mild in intensity (3–4 points on the VAS) at baseline. None of the patients had 9 or 10 pain intensity on the VAS initially.

The mean pain intensity in the pain flare was 8 (range 5–9). Out of 11 pain flare patients, in six of them (55%) there was an increase in pain on the visual analog scale after irradiation. All of them had an increase in pain of 2 points on the VAS. The other five patients (45%) reported “worse pain” following radiation and required analgesic escalation. All of them had a pain intensity of 8 at the baseline and reported pain of 9 on the VAS in the pain flare occurrence.

Dexamethasone or bisphosphonates as pain flare prophylaxis were not given to any patient. No statistically significant difference was observed in pain flare incidence regarding the type of analgesics used at the baseline (NSAIL, weak opioids, or strong opioids; Fisher exact test *p* = 0.924; *p* > 0.05).

Out of the eleven “pain flare” patients, in 9 patients (82%) it occurred after 48 h, in one after 24 h, and in one after 7 days of irradiation.

In the patient who had a pain flare in the first week after irradiation, the initial pain was 5 on the VAS, and the maximum was 7 points. The patient used NSAID analgesic therapy, and in the second and third week, a partial response (4 points on the VAS) was recorded without correction of the analgesic therapy.

During the first week after the irradiation, all patients had pain improvement. For pain flare management, in only one patient, dexamethasone was administered (a female patient with endometrial cancer and thoracic spine metastasis, initial pain intensity was 4 on VAS, and 6 in the flare). All others had only analgesic dose corrections.

No changes in ECOG PS were noted in any of the pain flare patients.

After the initial pain flare, five (45%) of the 11 patients achieved a complete pain response to the treatment and maintained that response until the conclusion of the study. Pain stabilization was achieved in five patients (45%), and with analgesic therapy, the pain did not increase further. Only one patient (10%) had a rapid progression of pain after initial stabilization (prostate cancer patient with pelvis metastasis). On the 4th week of follow‐up (1 month after radiotherapy), in our study, no statistically significant difference was observed in pain response between patients who had pain flare versus those who did not (Fisher exact test *p* = 0.877; *p* > 0.05).

In this group of patients, no statistically significant difference was observed in pain flare occurrence depending on the patient's age (Fisher exact test *p* = 0.962; *p* > 0.05), performance status (Fisher exact test *p* = 0.195; *p* > 0.05), the primary origin of the tumor, that is, the histological type of tumor (Fisher exact test *p* = 0.0995; *p* > 0.05), nor depending on the location of the metastasis (bone) that was irradiated (Fisher exact test *p* = 0.646; *p* > 0.05) (Table 3).

**TABLE 3:** Pain flare occurrence regarding the patient's age, performance status, primary origin, and localization of the metastases (bone) that were irradiated.

**Patients age**	**40–50 years**	**50–60 years**	**60–70 years**	**70–80 years**	**80–90 years**		
No pain flare	4 (100%)	12 (80.00%)	22 (78.57%)	11 (84.62%)	4 (100%)		
With pain flare	0 (0.00%)	3 (20.00%)	6 (21.43%)	2 (15.38%)	0 (0.00%)		
Total	4 (100%)	15 (100%)	28 (100%)	13 (100%)	4 (100%)		Fisher Exact Test: *p* = 0.962 (*p* > 0.05)
**Performance status**	**PS1**	**PS2**	**PS3**	**PS4**			
No pain flare	12 (66.67%)	16 (84.21%)	20 (90.91%)	5 (100%)			
With pain flare	6 (33.33%)	3 (15.79%)	2 (9.09%)	0 (0.00%)			
Total	18 (100%)	19 (100%)	22 (100%)	5 (100%)			Fisher Exact Test: *p* = 0.195 (*p* > 0.05)
**Primary origin**	**Breast**	**Gastro intestinal**	**Lungs**	**Gynecological**	**Unknown primary**	**Urological**	
No pain flare	5 (100%)	12 (100%)	18 (81.82%)	1 (33.33%)	3 (75.00%)	14 (77.78%)	
With pain flare	0 (0.00%)	0 (0.00%)	4 (18.18%)	2 (66.67%)	1 (25.00%)	4 (22.22%)	
Total	5 (100%)	12 (100%)	22 (100%)	3 (100%)	4 (100%)	18 (100%)	Fisher Exact Test: *p* = 0.0995 (*p* > 0.05)
**Localization of metastases irradiated**	**Spinal column**	**Pelvis**	**Femur + humerus**	**Rib**			
No pain flare	12 (75.00%)	31 (83.78%)	9 (90.00%)	1 (100%)			
With pain flare	4 (25.00%)	6 (16.22%)	1 (10.00%)	0 (0.00%)			
Total	16 (100%)	37 (100%)	10 (100%)	1 (100%)			Fisher Exact Test: *p* = 0.646 (*p* > 0.05)

## 4 Discussion

As modern therapy in oncology advances and develops, the life expectancy of oncological patients has been significantly extended, and the incidence of patients who will develop bone metastases during their lifetime is increasing. Therefore, the therapy of metastases and metastatic disease is of great clinical interest.

The pain that often accompanies bone metastases significantly impairs the patient's quality of life; thus, the therapy for painful bone metastases aims not only to relieve pain but to preserve function, mobility, and quality of life.

Radiotherapy is considered the standard treatment for localized, uncomplicated bone metastases with an overall response rate of 50%–90% and a complete response rate of 10%–50%. Since 1960, when Vargha et al. [13] published a result of 90% response to 18 Gy in one fraction applied to painful bone metastases, until today, prospective randomized studies worldwide have tried to establish the best dose–response ratio [14]. A 2017 meta‐analysis by Kougioumtzopoulou et al. concluded that there was no statistically significant difference in overall response to radiotherapy among different fractionation regimens but with a higher rate of retreatment after single‐fraction radiotherapy [15]. The Bone Trial Working Party Study Group (BTWPG) also showed no difference in time to pain relief, time to complete response, and time to pain progression during the 12‐month follow‐up of patients irradiated with 8 Gy in one fraction, 20 Gy in 5 fractions, or 30 Gy in 10 fractions. What's more, the difference was not observed either in the use of analgesics or in the toxic effects of radiation therapy [16, 17]. The conclusion that both single and multifractionated radiation regimens are equally effective in relieving pain met its final in a 2017 revision of the ASTRO guidelines for the treatment of painful bone metastases [41]. Analysis of the lowest effective dose of radiation in a single fraction across clinical studies has shown that 8 Gy is most likely the lowest optimal single shoot dose [12, 17].

During the COVID‐19 pandemic, 8 Gy single fraction became the preferred palliative radiotherapy regimen for symptomatic bone metastases since it was as efficient as it was time and cost‐effective [42].

Although recognized as an effective treatment in metastatic bone pain management, some authors reported single‐fraction radiotherapy to be particularly associated with a temporary worsening of bone pain in the irradiated site, known as pain flare [29, 30, 31]. Still, multi‐fraction radiotherapy and SRBT can cause this side effect nonetheless [23, 32, 33, 34].

The pain flare interferes with the patient's quality of life and performance status, leading to multiple hospital visits for analgesic dose corrections, which further leads to even more adverse events [24]. Who and why will develop pain flare remains largely unknown, leaving the physicians to deal with this phenomenon on a case‐to‐case basis. Until now, no consensus on the most effective treatment has been established [9, 25, 28, 43]. Based on the theory of inflammation and periosteal edema caused by radiation, the administration of corticosteroids (dexamethasone) in pain flare therapy and prophylaxis is suggested [26, 27].

Published literature data show significant variation in pain flare incidence ranging from 2% to 40% for EBRT. The authors speculate several reasons for this difference, such as inconsistent definition of pain flare, use of different radiotherapy regimens, and corticosteroid use, as well as the method of data collection. Retrospective studies are considered to be especially biased by patients' inability to accurately reconstruct past events [11, 33, 34]. With that in mind, our study was designed as prospective, 8 Gy single‐fraction only, with no prophylactic use of corticosteroids.

In our study, pain flare occurred in 11 out of 64 patients (17%) treated with 8 Gy single fraction radiotherapy for painful bone metastases. This result is somewhat lower compared to other authors. In the study of Chow et al. in 2005, pain flare incidence resembles ours with 2%–16% out of 88 patients included in the study [23]. However, later breakthrough studies (Gomez‐Iturriaga et al., Hird et al., Loblaw et al.) reported pain flare incidence to be about 40% for both 8 Gy single fraction and multi‐fraction groups of patients [11, 30, 34]. It should be noted that the conclusions about the pain flare incidence are challenging to withdraw since most of the studies include single fraction and multi‐fraction palliative radiotherapy protocols in the same pain flare study and refer to them later as a single pain flare cohort. Some of the studies concluded that patients receiving single‐fraction radiotherapy could be at higher risk for pain flare [29, 30, 31], but others failed to demonstrate that difference [23, 34].

The population of patients with painful bone metastases is very heterogeneous, and still approached for palliative radiotherapy as a single entity. Therefore, our group of patients was also heterogeneous, with more than a third of the patients having primary lung cancer, followed by prostate, breast, and colon. The previous studies were heterogeneous as well, including “any cancer” predominantly breast, lungs, and prostate as primary origin but with variable proportion.

For male to female ratio, our study is also following the literature (40% female and 60% male patients). Still, in the recent Egyptian pain flare study of Zaki et al. in 2021. There was female predominance with bone metastases of 58.1%, with breast cancer being the most predominant primary site [43]. The mean age in our group was 66 years (range 53–77) which fully corresponds to the global mean age of bone metastases of 64 years [43, 44]. Predominantly the metastases were localized on the spine and pelvis in our group of patients, which correlates with the most common reported metastatic sites.

The intensity of pain represents a very subjective and individual category, and that subjective feeling of the patient is taken as the gold standard for pain assessment since it is also considered the most reliable. In our study, the VAS scale was used to assess pain; the pain flare was defined according to the widely used Chow et al. definition, which is fully consistent with previously published studies.

In our group of patients who developed pain flare, there were seven male patients (64%) and four female patients (36%). The mean age in the pain flare group was 66 years (range 53–77). Lung cancer was the primary cancer origin in more than a third (37%) of the patients with pain flare. Two patients (18%) had renal cell cancer, two had gynecological tumors, one had prostate cancer, one had bladder cancer, and one had an unknown primary. In almost all of them, the metastases were localized in the spine and pelvis; only in one, the metastasis was in the humerus.

In 82% of our pain flare patients, it occurred after 48 h, in one after 24 h, and one after 7 days of irradiation. The mean pain intensity in the flare was 8 (range 5–9). Six (55%) out of 11 pain flare patients had an increase in pain on the VAS following radiotherapy (an increase in pain of 2 points on the VAS in all of them). The other five patients (45%) needed analgesic escalation for more than 25%, as defined. All of them had a pain intensity of 8 at the baseline and reported pain of 9 on the VAS in the pain flare occurrence. No statistically significant difference was observed in pain flare incidence regarding the type of analgesics used at baseline.

These results fully support current literature data that the pain flare is expected early (in the first days) after the irradiation. Most of the studies report pain flare incidence in the first 1–5 days [11, 34], but there is data about late pain flare occurrence (even 10 days after irradiation) [11]. Our results are in full consistency with other authors regarding the time of pain flare occurrence. However, Hird et al. reported a lower mean worst pain in flare being 6 (range 0–10) according to Brief Pain Inventory [34]; while the mean increase in pain of 2 points in the study of Chow et al. [23] corresponds to our results.

During the first week after the irradiation, all patients had pain improvement, which is following literature data stating a mean pain flare duration of 3 days [26]. For pain flare management, in only one patient, dexamethasone was administered, and all others had only analgesic dose corrections.

In our group of patients, no changes in ECOG PS were noted in any of the patients experiencing pain flare. The literature data on performance status change in flare is scarce.

After the initial pain flare, five of the 11 patients achieved complete pain response to the treatment and maintained that response until the conclusion of the study. Pain stabilization was achieved in five patients, and with analgesic therapy, the pain did not increase further. Only one patient had a rapid progression of pain after initial stabilization. No statistically significant difference was observed in pain response and pain stability between patients who had pain flare versus those who did not. These results fully support previous findings that pain flare does not predict response to treatment and that most of the pain flare patients show statistically significant improvement in pain control and performance status once the flare is withdrawn [11, 43].

The question of predictive parameters that might suggest a patient with a higher risk for pain flare occurrence still seems to be waiting for its answer. From a practical, clinical point of view, the identification of these patients would lead to a more personalized and optimized radiotherapy approach and tailored analgesic therapy, maintaining the quality of the patient's life and further oncological treatment.

Across the literature, there is very little and very heterogeneous data on the identification of patients with higher risk for developing pain flare both for EBRT and SBRT [11, 34, 45, 46]. In the single and multi‐fraction EBRT study of Gomez‐Iturriaga et al., no significant relation was found between the occurrence of pain flare and baseline variables such as gender type, primary cancer site, radiation dose, and radiation site [11]. Hird et al., however, found patients with primary breast cancer to be more prone to pain flare (52% in breast cancer, 25% in prostate cancer, and 23% in lung cancer) [34]. Even in the SBRT study of Pan et al., other than the fractionation regimen, no clinical parameters were found to influence the pain flare occurrence [45]. Chiang et al., on the other hand, found a higher Karnofsky performance score, cervical and lumbar spine metastatic location a significant predictor of pain flare in their SBRT study [46].

Analyzing our group of patients treated with single shoot radiotherapy for painful secondary bone deposits, no statistically significant difference was observed in the pain flare occurrence depending on the patient's age, general condition, primary origin of the tumor, that is, histological type of tumor, as well as depending on the location of the metastasis (bone) that was irradiated. These results are in full correspondence with the study of Gomez‐Iturriaga et al.

Our study, however, has several limitations. With a modest sample size of 64 heterogeneous patients (heterogeneous primary tumor site, histology, bone metastases localization, etc.) with an even smaller proportion of patients having pain flare (11 patients, 17%), it is challenging to identify predictive factors for this phenomenon; further studies are warranted.

From the broader radiation oncology perspective, it is even more complex. Since novel radiotherapy techniques such as SBRT are increasingly in use in selected patients with painful bone metastases, questions of safety, feasibility, and optimization of the technique selection are rising; encouraging even more research [32, 47].

According to scarce and heterogeneous available data, and the limitations of our as well as other authors' studies, it seems that at this point we do not yet have a clear image of a pain flare‐prone patient.

## 5 Conclusion

Based on the results of our study, pain flare can be expected in less than 20% of patients receiving 8 Gy single fraction external‐beam radiotherapy for painful bone metastases. It is not proven that any of the mentioned clinical parameters influence the occurrence of pain flare. However, pain flare does not interfere with response to treatment; significant improvement in pain control and performance status can be achieved with a “simple single shoot”. Further, larger, prospective studies are of great importance to address the problem of pain flare incidence discrepancy and to identify the patients with greater risk for this disturbing phenomenon.

## Author Contributions

**Tatjana Arsenijević:** conceptualization (equal), data curation (lead), formal analysis (lead), investigation (lead), methodology (equal), writing – original draft (lead). **Aleksandar Stepanović:** data curation (equal), writing – review and editing (equal). **Snježana Bogićević:** data curation (equal). **Dragica Gržetić:** data curation (equal). **Marina Nikitović:** conceptualization (equal), methodology (equal), writing – review and editing (equal).

## Ethics Statement

The study was conducted according to the guidelines of the Declaration of Helsinki. Ethical review and approval were waived in this study since no experiments or novel treatments were conducted on humans (patients). The methodology represents a standard treatment modality that does not need ethical approval. The decision to treat the patients in the manner described in the study was made by the multidisciplinary tumor board of the Institute for Oncology and Radiology of Serbia. The manuscript is a part of the academic sub‐specialistic thesis of T.A. approved by the Faculty of Medicine, University of Belgrade, Serbia, 2019 (Protocol number: 04 BR: 18‐UON‐15).

## Consent

Written informed consent was obtained for all subjects involved in the study. All subjects were fully aware that the data would be published. Written informed consent included that statement too. No personal identifiers are presented in this study.

## Conflicts of Interest

The authors declare no conflicts of interest.

## Data Availability

All data is contained within the article. Any additional information or explanation is available from the corresponding author.
